# Validation of different staging systems for hepatocellular carcinoma in a cohort of 249 patients undergoing radiotherapy

**DOI:** 10.18632/oncotarget.14881

**Published:** 2017-01-28

**Authors:** Zhi-Rui Zhou, Min Liu, Hui-Rong Lu, Ye-Fei Li, Shi-Xiong Liang, Chun-Yan Zhang

**Affiliations:** ^1^ Department of Radiation Oncology, Fudan University Shanghai Cancer Center, Shanghai, P.R. China; ^2^ Department of Oncology, Shanghai Medical College, Fudan University, Shanghai, P.R. China; ^3^ Department of Radiation Oncology, Cancer Hospital of Guangxi Medical University, Nanning, Guangxi Zhuang Autonomous Region, P.R. China; ^4^ Department of Radiation Oncology, Shanghai Pulmonary Hospital, Tongji University School of Medicine, Shanghai, P.R. China; ^5^ Department of Experimental Research, Cancer Hospital of Guangxi Medical University, Nanning, P.R. China

**Keywords:** hepatocellular carcinoma, radiotherapy, cancer staging

## Abstract

There is no consensus on predicting prognosis for hepatocellular carcinoma patients undergoing radiotherapy. This study aims to evaluate the validity of different staging systems. Overall, 249 hepatocellular carcinoma patients were evaluated retrospectively. All patients were classified by different staging systems. The cumulative survival rates were calculated using the Kaplan-Meier method, and survival curves were compared using the log-rank test. Harrell's concordance index (c-index) was calculated. The 1-, 3-, and 5-year overall survival rates were 58%, 31% and 20%, respectively. Significant differences in overall survival were observed between stages I and II of the Okuda staging system (*p*=0.004), between scores of 3 and 4 of Cancer of the Liver Italian Program prognostic score (*p*=0.009), between Chinese University Prognostic Index low-risk and intermediate-risk groups (*p*=0.01), between 1 and 2 points of the Japan Integrated Staging score (*p*=0.037), between stages III and IV of American Joint Committee on Cancer 1997 TNM staging system (*p*=0.011), between stages II and III of American Joint Committee on Cancer 2002 TNM staging system (*p*=0.026) and between stages I and II of Guangzhou 2001 staging system (*p*=0.000). In conclusion, the Okuda staging system, Chinese University Prognostic Index, and Chinese Guangzhou 2001 staging system were more discriminative than the other staging systems in the prognostic stratification for hepatocellular carcinoma patients undergoing radiotherapy.

## INTRODUCTION

Hepatocellular carcinoma (HCC) was the fourth leading cause of global cancer deaths in 2012 [1–3]. Depending on the extent of the disease and the underlying liver status, patients may be treated with local, locoregional, and/or systemic therapy. Several clinical trials have indicated that radiotherapy (RT) can play a meaningful role in the management of HCC [4–8]. Although there is no high level evidence from randomized controlled trial, the efficacy and safety of RT in HCC has been shown by several prospective or retrospective trials using modern RT techniques, especially in Asia-Pacific, where have higher incidence of HCC than other areas [9, 10]. In the 5th Asia-Pacific Primary Liver Cancer Expert Meeting (APPLE 2014), a consensus on the utility and efficacy of RT in the treatment of HCC according to different cancer stage have been made: in early and intermediate stage HCC, if standard treatment is not compatible, RT, including EBRT and SIRT can be considered. In locally advanced stage HCC, combined EBRT with transarterial chemoembolization or hepatic arterial infusion chemotherapy, and SIRT can be considered [9].

The goals of cancer staging are to predict prognosis and to provide therapeutic guidelines; they are also useful to stratify patients enrolled in clinical trials. A variety of new HCC staging systems are emerging. There are currently 10 different staging systems relevant to HCC. These include the Okuda staging system [11], Cancer of the Liver Italian Program (CLIP) prognostic score [12, 13], Barcelona Clinic Liver Cancer (BCLC) classification system [14], France staging system [15], Chinese University Prognostic Index (CUPI) [16], Japan Integrated Staging (JIS) score [17], American Joint Committee on Cancer (AJCC) 1997 Tumor-Node-Metastasis (TNM) staging system [18], AJCC 2002 TNM staging system [19], AJCC 2010 TNM staging system [20] and the Chinese Guangzhou 2001 staging system [21]. No other single cancer has such a large number of proposed staging systems, which reflects a general sense that the current HCC staging systems are less than satisfactory. Furthermore, there are even more newly proposed staging systems for HCC patients.

However, few staging systems are based on radiotherapy. To date, only Jensen Seong et al. have addressed this [22]. Most existing staging systems are proposed based on surgery rather than radiotherapy. Therefore, the purpose of our study was to assess the validity of the 10 staging systems in HCC patients receiving radiotherapy in our cohort.

## RESULTS

### Baseline characteristics and 249 HCC patient distribution by staging system

A total of 249 patients were included in the analyses; their performance status and liver function are shown in Table 1. The patients’ disease classifications based on different cancer staging systems are shown in Table 2. More than 50% of patients were classified as Okuda stage I, BCLC stage C, France stage B, CUPI low-risk group, JIS score 1, AJCC 1997 TNM staging system stage T1, AJCC 2002 TNM staging system stage T1, AJCC 2010 TNM staging system stage T1 and Guangzhou 2001 stage II. No patient was classified as CLIP score 5, France stage C, JIS score 0, 4, 5. According to the Okuda staging and CUPI, 95% of patients were classified as early and middle stage. Among 249 patients, the median survival time was 15 months. The overall survival rates at 1, 2, 3, 4 and 5 years were 58%, 37%, 31%, 25% and 20%, respectively.

**Table 1:** Clinical characteristics of 249 eligible HCC patients undergoing RT

Variables	No. of patients	%
Age (year)		
Median(range)	48(23-82)	
Sex (Male)	218	87.6
Symptoms (Yes)	208	83.5
Abdominal pain (Yes)	167	67.1
Weight loss (Yes)	58	23.3
Alcohol (Yes)	64	25.7
HBV (Positive)	205	82.3
Cirrhosis (Yes)	145	58.2
Splenomegaly (Yes)	83	33.3
Tumor size (≤5cm)	48	19.3
Tumor number		
1	214	85.9
≥2	35	14.1
AFP(>400ng/ml)	143	57.4
Tumor thrombosis (Yes)	69	27.7
Vascular invasion (Yes)	99	39.8
Total dose of RT		
Median(range)	52Gy(32Gy-72Gy)	
Fraction dose of RT		
Median(range)	4.6Gy(1.8Gy-8Gy)	
Total times of RT		
Median(range, day)	12(6-35)	
Times per week		
3	220	88.4
4	19	7.6
5	10	4
Interventional therapy	154	61.8

**Table 2:** 249 HCC patient distribution by different staging systems

Staging systems	Stage	No. of patients	%
The Okuda Staging System	I II III	171 77 1	68.7 30.9 0.4
Cancer of the Liver Italian Program prognostic score	0 1 2 3 4	46 78 55 49 21	18.5 31.2 22.1 19.7 8.4
Barcelona Clinic Liver Cancer Classification System	A B C	8 23 218	3.2 9.2 87.6
The France Staging System	A B	19 230	7.6 92.4
Chinese University Prognostic Index	Low-risk group Intermediate-risk group High-risk group	168 78	67.5 31.3
Japan Integrated Staging	1 2 3	3 127 110	1.2 51 44.2
TNM staging system (1997)	T1 T2 T3 T4	12 2 125 18	0.8 0.8 50.2 7.2
TNM staging system (2002)	T1 T2 T3 T4	104 127 13 101	41.8 51 5.2 40.6
TNM staging system (2010)	T1 T2 T3 T4	8 127 13 101	3.2 51 5.2 40.6
Guangzhou2001	I II III	8 40 185 24	3.2 16.1 74.3 9.6

### Survival analysis by the Okuda staging system

**Figure 1:**
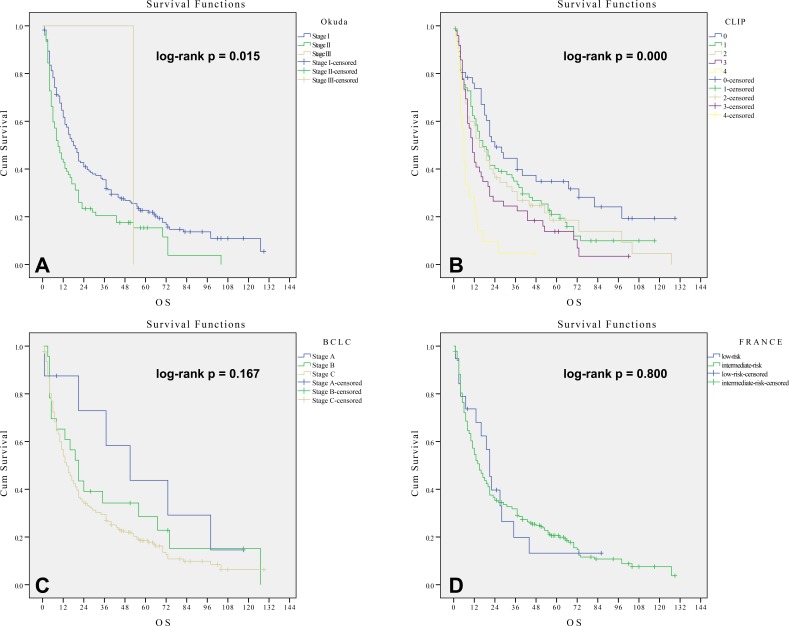
**Kaplan-Meier method estimates of cumulative survival curves by the Okuda staging system A., CLIP prognostic score B., BCLC classification system C. and France staging system D. in 249 patients undergoing RT**.

The median survival times for patients with Okuda stages I, II and III were 18, 9, 53 months, respectively (log-rank *p value* = 0.015; C-index = 0.5504, standard error (se) = 0.0181). As shown in Figure 1A, Okuda stage I patients had a significantly longer survival than those patients with Okuda stage II (*p value* = 0.004). Because only one patient was classified as Okuda stage III, the difference between stage II and stage III could not be evaluated. For patients with early and middle stage HCC who underwent radiotherapy, the Okuda staging system predicted survival well in this cohort.

### Survival analysis by the CLIP prognostic score

The median survival times for CLIP scores 0, 1, 2, 3 and 4 were 24, 17, 15, 11 and 6 months, respectively (log-rank *p value* = 0.000; C-index = 0.5834, se = 0.0225). As shown in Figure 1B, there was no significant difference in survival among patients with CLIP scores 1 to 3 points (*p* > 0.05). Significant differences in OS were observed when comparing patient groups with CLIP scores of 0, 3 and 4 (*p* = 0.009). Therefore, the CLIP prognostic score shows some limitations in predicting prognosis in this cohort.

### Survival analysis by BCLC classification system

The median survival times for BCLC stages A, B and C were 51, 21 and 14 months, respectively (log-rank *p value* = 0.167; C-index = 0.5225, se = 0.0138). As shown in Figure 1C, there was no significant difference in OS between patients with BCLC stages A and B or between patients with BCLC stages B and C (*p* > 0.05). Therefore, the BCLC classification system had no discriminative power in predicting prognosis in this cohort.

### Survival analysis by France staging system

The median survival times for the France low-risk group and intermediate-risk group were 21 and 15 months, respectively (log-rank *p value* = 0.800; C-index = 0.5050, se = 0.0108). There was no significant difference in OS between the low-risk and intermediate-risk groups (*p* > 0.05), Figure 1D. This result suggested that the France staging system had no discriminative power in stratifying patients and predicting prognosis in this cohort.

### Survival analysis by Chinese university prognostic index

**Figure 2:**
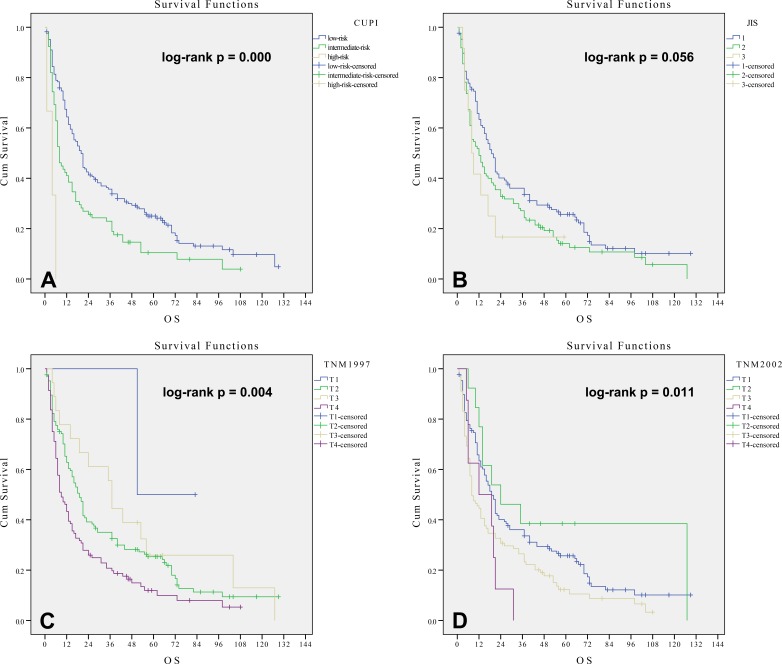
**Kaplan-Meier method estimates of cumulative survival curves by the CUPI system A., JIS system B., TNM 1997 system C. and TNM 2002 system D. in 249 patients undergoing RT**.

The median survival times for the CUPI low-risk group, intermediate-risk group and high-risk group were 20, 8 and 4 months, respectively (log-rank *p value* = 0.000; C-index = 0.5746, se = 0.0181). As shown in Figure 2A, CUPI low-risk group patients had a significantly longer survival than did intermediate-risk group patients (*p* = 0.01). CUPI intermediate-risk group patients had a significantly longer survival than did high-risk group patients (*p* = 0.011). For HCC patients undergoing radiotherapy, CUPI was a good predictor of overall survival in this cohort.

### Survival analysis by Japan integrated staging

The median survival times for JIS scores 1, 2 and 3 were 19, 12 and 8 months, respectively (log-rank *p value* = 0.056; C-index = 0.5495, se = 0.0204). There was no significant difference in OS between scores of 2 and 3 points (*p* > 0.05). A difference was observed when comparing patient groups with JIS scores of 1 and 2 points (*p* = 0.037), Figure 2B. Therefore, JIS shows some limitations in predicting prognosis in this cohort.

### Survival analysis by AJCC 1997 TNM staging system

The median survival times for AJCC 1997 TNM stages T2, T3 and T4 were 19, 37 and 9 months, respectively (log-rank *p value* = 0.004; C-index = 0.5649, se = 0.0208). Only the difference in OS between stages T3 and T4 was significant (*p* = 0.011), with the median survival time of T3 longer than that of T2. This result is not common sense, as shown in Figure 2C. The AJCC 1997 TNM staging system had no discriminative power in stratifying patients and predicting prognosis in this cohort.

### Survival analysis by AJCC 2002 TNM staging system

The median survival times for AJCC 2002 TNM stages T1, T2, T3 and T4 were 19, 24, 8 and 12 months, respectively (log-rank *p value* = 0.011; C-index = 0.5578, se = 0.0207). Stage T2 patients had significantly longer survival times than stage T3 patients (*p* < 0.05). However, there was no significant difference in OS either among patients with T1 and T2 or among patients with T3 and T4 (*p* > 0.05). In addition, the median survival time of T2 was longer than that of T1 and was longer for T4 than for T3, Figure 2D. The AJCC 2002 TNM staging system could not predict prognosis well in this cohort.

### Survival analysis by AJCC 2010 TNM staging system

**Figure 3:**
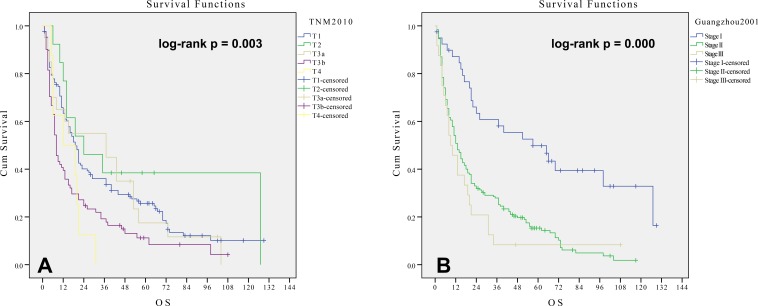
**Kaplan-Meier method estimates of cumulative survival curves by the TNM 2010 A. and Chinese Guangzhou 2001 staging system B. in 249 patients undergoing RT**.

The median survival times for AJCC 2010 TNM stages T1, T2, T3a, T3b and T4 were 19, 24, 37, 8 and 12 months, respectively (log-rank *p value* = 0.003; C-index = 0.5673, se = 0.0210). T1 patients had shorter median survival times than did T2 and T3a patients, and T3a patients had the longest median survival time. Obviously, this is not common sense. As shown in Figure 3A, the AJCC 2010 TNM staging failed to predict prognosis in the HCC patients in this cohort.

### Survival analysis by the Chinese Guangzhou 2001 staging system

The median survival times for Guangzhou 2001 stages I, II and III were 57, 13 and 12 months, respectively (log-rank *p value* = 0.000; C-index = 0.5789, se = 0.0182). As shown in Figure 3B, Guangzhou 2001 stage I patients had a significantly longer survival than stage II (*p* = 0.000). Patients with stages II and III had similar survival times (*p* = 0.301). For early and intermediate stage HCC patients undergoing radiotherapy, the Guangzhou 2001 staging system was a good predictor of overall survival.

## DISCUSSION

The Okuda staging system was one of the first developed [11]. Although it does not consider tumor factors in detail, it does account for liver function status, which plays an important role in predicting prognosis [15]. In our study, Okuda stage I patients had a significantly longer survival than Okuda stage II patients (*p* = 0.004). Although the difference between stages II and III could not be evaluated because only one patient was classified as Okuda stage III, the Okuda staging system was successful in predicting survival for early and intermediate stage HCC patients.

The CLIP prognostic score accounts for liver function status and detailed tumor factors [12, 13]. However, several retrospective analyses showed that a significant difference was present among some stages. Levy found that the CLIP prognostic score can identify the patients with a significantly better prognosis or a significantly worse prognosis, when he studied on the prognosis of 257 HCC patients [23]. Our study showed similar results; survival differences were found only among CLIP scores 0, 3 and 4 (*p* = 0.009). The result of our study is similar to that of Korea [22]. The assessment of prognosis using the CLIP prognostic score needs improvement.

The BCLC [14] classification system was reported to be the best therapeutic guideline, especially for early liver cancer patients who can receive radical surgery [23, 24]. However, it had some deficits: (1) scoring is complex and requires more parameters; and (2) it is not feasible to obtain information on values such as performance score (PS) and portal hypertension for a retrospective clinical study. Our studies showed that there was no statistical differentiation among all stages.

The variables of France staging system [15] are liver function status, performance status test (PST) and portal vein cancerous thrombosis. Both it and the BCLC classification system include subjective factors such as PST. Our study showed that there was no significant difference in overall survival between the low- and intermediate-risk groups (*p* = 0.800).

Our study found that CUPI [16] was good at predicting overall survival for HCC patients undergoing radiotherapy. CUPI was constructed for patients with HCC based on large numbers of patients. However, almost all of the patients had HBV-related HCC (79%). Europe and the United States populations are more typically infected with the HCV virus, while the Asian population is more commonly infected with the HBV virus; perhaps it can be said that CUPI applies to patients with HBV-related liver cancer to predict the prognosis of overall survival. This study found that CUPI was able to better predict patient survival prognosis that could not be excluded precisely because of this feature; an American scholar demonstrated that CUPI showed a better prognostic ability with HCC patients undergoing transcatheter arterial chemoembolization (TACE) [25]. The limitations of our study were mainly due to the unbalanced distribution of different stage HCC patients, which makes it impossible to compare all patients. Therefore, further studies should expand the sample size to balance patients among different stages.

The JIS scoring system [17] was proposed based on the integrated Japanese TNM staging system and Child-Pugh class. The Liver Cancer Study Group of Japan indicated that the JIS scoring system was more discriminative than Japanese TNM. However, a separate trend of Kaplan-Meier survival curves between JIS scores 1 and 2 was not obvious. Hence, JIS shows some limitations in predicting prognosis.

The AJCC TNM staging system was considered the best staging system in solid tumors and was adopted for clinical practice. The AJCC 1997 TNM staging system [18] indicated that some tumor features such as tumor size, tumor number, vascular infusion, lymph node metastasis and distant metastasis had the same prognostic value. However, some studies have demonstrated that they were not all the same [26, 27]. The AJCC 2002 TNM staging system lacked liver function status; the AJCC 2002 TNM staging system then combined liver function scoring to enhance the prognostic ability [19]. However, in our study, all AJCC TNM staging systems were far from satisfactory. Vauthey LN and the International Collaborative Group obtained similar results [19, 25]. The AJCC TNM staging system had low approval for the following reasons: (1) lack of liver function status; (2) difficult to clear vascular infusion, especially microvascular infusion; and (3) difficult to compare and evaluate because each edition of AJCC TNM staging system changed tremendously.

The Guangzhou 2001 staging system was based on hepatectomy [21, 28]. It is debatable whether it is valid regarding treatment strategy beyond surgery. Some Chinese scholars considered the Guangzhou 2001 staging system to be more suitable for Chinese HCC patients. Our study showed that a separate trend of Kaplan-Meier survival curves between stages I and II was extremely obvious. For early and intermediate HCC patients undergoing radiotherapy, the Guangzhou 2001 staging system did well in predicting overall survival in HCC patients undergoing radiotherapy.

However, the manuscript has the following limitations: First, it is a retrospective study. In this retrospective analysis, we only consider the different clinical stage, not fully consider all possible confounding factors that potentially impact on patients’ overall survival, such as other treatment methods before or after radiotherapy. But, we think that it does not badly affect our results. Take Okuda staging system for example, total 88 HCC patients received transcatheter arterial chemoembolization (TACE), the proportion of patients received TACE is balanced between Okuda stage 1 and stage 2 (*p* = 0.757). Besides radiotherapy or TACE, a few patients have received the biotherapy and other treatment. The diversity of treatments may introduce unpredictable confounding factors. Additionally, only Child-Pugh A HCC patients were included in this study, which further limits the representativeness of the conclusion. In fact, all of the C-index were too small, with limited value, it well proved that the existing staging systems for liver cancer are flawed, and need to be improved.

In conclusion, our study demonstrated that the Okuda staging system, CUPI and the Chinese Guangzhou 2001 staging system were more discriminate than the other staging systems in prognostic stratification for early and middle stage HCC patients treated with radiotherapy. The others (CLIP prognostic score, BCLC classification system, the France staging system, JIS score, AJCC 1997 TNM staging system, AJCC 2002 TNM staging system and AJCC 2010 TNM staging system) performed poorly in stratifying the liver cancer patients undergoing radiotherapy in our study.

## METHODS AND MATERIALS

### Patients

Between April 1999 and March 2012, 249 eligible liver cancer patients who had not undergone curative resection were identified from the patient files of the Cancer Hospital of Guangxi Medical University and were included in the study. Patients with HCC were diagnosed based on the clinical diagnostic criteria proposed by the Chinese Society of Liver Cancer and the Chinese Anti-Cancer Association (CACA) in 2011 [28] or based on a pathologic diagnosis. Patients with primary HCC received radiotherapy using the three-dimensional conformal radiation therapy (3-DCRT) technique as the main treatment approach were included. Total dose of RT should be more than 30 Gy, Fraction dose of RT should not be less than 1.8 Gy/fraction. Patients with cirrhosis of the liver have been identified as being an independent risk for HCC, and HCC is the principal cause of death in patients with cirrhosis [29, 30], so liver cancer patients with cirrhosis were included in the study. The Child-Pugh Grade was also an independent prognostic factor for HCC [4]. We only included HCC patients with Child-Pugh Grade A in our study, considering the potential influence of the Child-Pugh Grade [31]. Whether Child-Pugh Grade B patients can accept radiation therapy is controversial, Child-Pugh Grade C is not suitable for radiotherapy. Thus, Child-Pugh Grade B and Child-Pugh Grade C HCC patients were excluded. Patients with extrahepatic metastasis were excluded. This study was approved by the Institutional Review Board of Cancer Hospital of Guangxi Medical University, which waived the requirement for informed consent. The abstract of this paper was presented as a poster at the American Society for Radiation Oncology 56th Annual Meeting, October 28-31, 2012, in Boston, Massachusetts.

### Staging criteria

Two experienced reviewers independently reviewed the stages for all eligible patients following different staging systems and resolved controversies by discussion. The 10 staging systems included the Okuda staging system, Cancer of the Liver Italian Program (CLIP) prognostic score, Barcelona Clinic Liver Cancer (BCLC) classification system, the France staging system, Chinese University Prognostic Index (CUPI), Japan Integrated Staging (JIS) score, American Joint Committee on Cancer (AJCC) 1997 Tumor-Node-Metastasis (TNM) staging system, AJCC 2002 TNM staging system, AJCC 2010 TNM staging system and the Chinese Guangzhou 2001 staging system. Data were collected to compare the validity of the different staging systems in HCC patients undergoing 3-DCRT.

Tumor morphology was determined based on computerized tomography (CT). Vascular invasion was assessed using dynamic CT and angiography. The maximum diameter of the tumor was measured using CT or ultrasonography. Lymph node metastasis and distant metastases were assessed using conventional anatomic imaging, such as ultrasound, dynamic CT scans and X-rays.

### Treatment

All patents underwent a planning CT scan to facilitate three-dimensional treatment planning using the Topslane planning system (Topslane Medical Corp., Shanghai, China) or the Precise planning system (Elekta Corp., Sweden). The gross volumes (GTV) were defined as the radiographically abnormal areas presented on the diagnostic CT and/or magnetic resonance imaging. The planning target volume (PTV) was defined by adding 0.5-1.5 cm to GTV. Liver motion was considered, with additional margins added to account for motion. The fraction sizes and total doses were determined based on the individual physician's professional judgment.

### Follow up

Patients were followed up every 3 months for 1 year and then every 6 months after treatment until death or the termination of this study (March 2016). The date of death was obtained from either patient records or death registers or family dependents. In patients with local recurrences of the primary lesion or with development of new lesions, a new treatment strategy was usually planned and performed. Length of survival was calculated from the date when the radiotherapy commenced.

### Statistical analysis

All statistical analyses were performed with SPSS version 16.0 (SPSS Inc., Chicago, IL, USA) and R (version: 3.3.1). The cumulative survival rate was calculated using the Kaplan-Meier method. Survival curves were statistically compared using the log-rank test; univariate cox regression was performed and the Harrell's concordance index (c-index) was calculated in the R software. A two-tailed *p* < 0.05 was considered statistically significant.

## SUPPLEMENTARY TABLE


