# Supplementary material for: Validation of different staging systems for hepatocellular carcinoma in a cohort of 249 patients undergoing radiotherapy
Source: Oncotarget. 2017 Jan 28;8(28):46523–31. doi: 10.18632/oncotarget.14881 (PMC5542288; doi:10.18632/oncotarget.14881)
Supplement: Supplementary file 1 [file oncotarget-08-46523-s001.doc]

Okuda staging system

|  | score | |
| --- | --- | --- |
|  | 0 | 1 |
| Bilirubin(mg/dl)  Albumin(g/dl)  Ascites  Tumor size(% of liver) | <3.0  ≥3.0  No  <50% | >3.0  <3.0  Yes  >50% |

CLIP scoring system

| Variables | score | | |
| --- | --- | --- | --- |
|  | 0 | 1 | 2 |
| Child-Pugh class  Tumor morphology  AFP（ng/dl）  Portal vein thrombosis | A  ≤50% of liver  <400  No | B  ≤50% of liver  ≥400  Yes | C  >50% of liver |

**BCLC staging system**

| Stage | PST | tumor statue | Okuda stage | Liver function Status |
| --- | --- | --- | --- | --- |
| A |  |  |  |  |
| A1 | 0 | Single,<5cm | I | No portal hypertension and normal bilirubin |
| A2 | 0 | Single,<5cm | I | portal hypertension and normal bilirubin |
| A3 | 0 | Single,<5cm | I | portal hypertension and abnormal bilirubin |
| A4 | 0 | 3 tumors <3cm | I-II | Child-Pugh A-B |
| B | 0 | Large multinodular | I-II | Child-Pugh A-B |
| C | 1-2 | Vascular invasion or extrahepatic spread | I-II | Child-Pugh A-B |
| D | 3-4 | Any | III | Child-Pugh C |

France staging system

| Weight | 0 | 1 | 2 | 3 |
| --- | --- | --- | --- | --- |
| KPS  bilirubin(umol/l)  ALP(IU/L)  AFP(ug/l)  Portal obstruction | ≥80  <50  <200  <35  No | Yes | ≥200  ≥35 | <80  ≥50 |

CUPI

| variable | Weigh(CUPI score) |
| --- | --- |
| TNM stage  I and II  IIIa and IIIb  Iva and IVb  Asymptomatic disease on presentation  Ascites  AFP≥500ng/ml  TB(umol/L)  <34  34-51  ≥52  ALP≥200 IU/L | -3  -1  0  -4  3  2  0  3  4  3 |

Japanese TNM staging system

| Factors  Number of tumors:single  Tumor size: <2cm  No vessel invasion(portal vein,hepatic vein,bile duct) | |
| --- | --- |
| T1  T2  T3  T4  N  Stage I  Stage II  Stage III  Stage IVa  Stage IVb | Fulfilling all three factors  Fulfilling two factors  Fulfilling one factor  Fulfilling none of the factors  Regional  T1N0M0  T2N0M0  T3N0M0  T4N0M0 or TxN1M0  TxNxM1 |

JIS scoring system

| Variable |  | Score |  |
| --- | --- | --- | --- |
|  | 0 | 1 | 2 |
| Japanese TNM staging system  Child-Pugh | I  A | II  B | III  C |

AJCC 1997 TNM Staging System

| Primary Tumor（T）  Tx:Primary tumor cannot be assessed  T0:No evidence of primary tumor  T1:Single tumor,≤2cm，, without vascular invasion  T2: Single tumor,≤2cm，with vascular invasion ; multiple tumors in one lobe,none>2cm, without vascular invasion;single tumor, >2cm, without vascular invasion  T3:Single tumor, >2cm, with vascular invasion; multiple tumors in one lobe,none>2cm, with vascular invasion; multiple tumors in one lobe,any>2cm, with or without vascular invasion.  T4:Multiple tumors in more than one lobe;tumor involving major branch of portal or hepatic vein;invasion of adjacent organs other than gallbladder  Regional Lymph Node(N)  Nx:Regional lymph nodes cannot be assessed  N0:No regional lymph node metastasis  N1: Regional lymph node metastasis  Distant Metastasis  Mx:Distant metastasis cannot be assessed  M0: No distant metastasis  M1: Distant metastasis  Stage Grouping  Stage I:T1N0M0  Stage II:T2N0M0  Stage IIIa:T3N0M0  Stage IIIb:T1-3N1M0  Stage IVa:T4NxM0  Stage IVb:TxNxM1 |
| --- |

AJCC 2002 TNM Staging System

| Primary Tumor（T）  Tx:Primary tumor cannot be assessed  T0:No evidence of primary tumor  T1:Solitary tumor without vascular invasion  T2:Solitary tumor with vascular invasion or multiple tumors none more than 5cm  T3:Multiple tumors more than 5cm or tumor involving a major branch of the portal or hepatic vein  T4:Tumor with direct invasion of adjacent organs other than the gallbladder or with perforation of visceral peritoneum  Regional Lymph Node(N)  Nx:Regional lymph nodes cannot be assessed  N0:No regional lymph node metastasis  N1: Regional lymph node metastasis  Distant Metastasis  Mx:Distant metastasis cannot be assessed  M0: No distant metastasis  M1: Distant metastasis  Stage Grouping  Stage I:T1N0M0  Stage II:T2N0M0  Stage IIIa:T3N0M0  Stage IIIb:T4N0M0  Stage IIIc:TxN1M0  Stage IV:TxNxM1 |
| --- |

AJCC 2010 TNM Staging System

| Primary Tumor（T）  Tx:Primary tumor cannot be assessed  T0:No evidence of primary tumor  T1:Solitary tumor without vascular invasion  T2:Solitary tumor with vascular invasion or multiple tumors none more than 5cm  T3a:Multiple tumors more than 5cm  T3b: Tumor involving a major branch of the portal or hepatic vein  T4:Tumor with direct invasion of adjacent organs other than the gallbladder or with perforation of visceral peritoneum  Regional Lymph Node(N)  Nx:Regional lymph nodes cannot be assessed  N0:No regional lymph node metastasis  N1: Regional lymph node metastasis  Distant Metastasis  Mx:Distant metastasis cannot be assessed  M0: No distant metastasis  M1: Distant metastasis  Stage Grouping  Stage I:T1N0M0  Stage II:T2N0M0  Stage IIIa:T3An0M0  Stage IIIb: T3Bn0M0  Stage IIIc: T4N0M0  Stage Iva:TxN1M0  Stage Ivb:TxNxM1 |
| --- |

Guangzhou 2001 stging system

| Ia:Single tumor,≤3cm,without tumor thrombosis,abdominal lymph node metastasis and distant metastasis,Child-Pugh A  Ib: Single tumor or two tumors ≤5cm,in half of liver,without tumor thrombosis,abdominal lymph node metastasis and distant metastasis,Child-Pugh A  IIa: Single tumor or two tumors ≤10cm,in half of liver,or two tumors ≤5cm,in different half of liver ,without tumor thrombosis,abdominal lymph node metastasis and distant metastasis,Child-Pugh A  IIb：Single tumor or two tumors >10cm,in any half of liver,or two tumors >5cm,in different half of liver ,without tumor thrombosis,abdominal lymph node metastasis and distant metastasis,Child-Pugh A;any tumor morphology,and tumor involving one of a branch of the portal or hepatic vein or with Bile duct tumor thrombus or/and Child-Pugh B  IIIa:Any tumor morphology, tumor involving one of a major branch of the portal vein or with Inferior vena cava tumor thrombus or with abdominal lymph node metastasis or with distant metastasis Child-Pugh A or B  IIIb:Any tumor morphology, tumor thrombus and metastasis,Child-Pugh C. |
| --- |
